# Progression of Neuronal Damage in an *In Vitro* Model of the Ischemic Penumbra

**DOI:** 10.1371/journal.pone.0147231

**Published:** 2016-02-12

**Authors:** Joost le Feber, Stelina Tzafi Pavlidou, Niels Erkamp, Michel J. A. M. van Putten, Jeannette Hofmeijer

**Affiliations:** 1 Clinical Neurophysiology, MIRA Institute for Biomedical Technology and Technical Medicine, University of Twente, Enschede, The Netherlands; 2 Department of Clinical Neurophysiology, Medisch Spectrum Twente, Enschede, The Netherlands; 3 Department of Neurology, Rijnstate Hospital, Arnhem, The Netherlands; Massachusetts General Hospital/Harvard Medical School, UNITED STATES

## Abstract

Improvement of neuronal recovery in the ischemic penumbra around a brain infarct has a large potential to advance clinical recovery of patients with acute ischemic stroke. However, pathophysiological mechanisms leading to either recovery or secondary damage in the penumbra are not completely understood. We studied neuronal dynamics in a model system of the penumbra consisting of networks of cultured cortical neurons exposed to controlled levels and durations of hypoxia. Short periods of hypoxia (pO_2_≈20mmHg) reduced spontaneous activity, due to impeded synaptic function. After ≈6 hours, activity and connectivity partially recovered, even during continuing hypoxia. If the oxygen supply was restored within 12 hours, changes in network connectivity were completely reversible. For longer periods of hypoxia (12–30 h), activity levels initially increased, but eventually decreased and connectivity changes became partially irreversible. After ≈30 hours, all functional connections disappeared and no activity remained. Since this complete silence seemed unrelated to hypoxic depths, but always followed an extended period of low activity, we speculate that irreversible damage (at least partly) results from insufficient neuronal activation. This opens avenues for therapies to improve recovery by neuronal activation.

## Introduction

The clinical impact of acute ischemic stroke is directly related to the severity of remaining neurological impairment [1]. The only treatment of proven benefit to reduce neurological impairment is acute re-canalization by intravenous thrombolysis [2] or intra-arterial thrombectomy [3]. The sooner this treatment can be administered, the better the prognosis, reflecting the vulnerability of the brain to ischemia [2,3]. In the subacute phase, improvement of neuronal recovery in the ischemic penumbra has a large potential to advance clinical recovery. However, current treatments to promote recovery of penumbral tissue are scarce and only consist of general interventions, such as prevention of pyrexia, hyperglycemia, and systemic hypotension. Moreover, when secondary damage of penumbral tissue leads to additional neurological impairment, which occurs in approximately one third of patients during the first days after the infarct, there is no therapy available [1].

In the infarct core, where perfusion levels drop below 10 ml/100g/min, energy supply is insufficient to preserve ion gradients across the plasma membrane [4]. Loss of neuronal function is followed by ion influx, cell swelling, and neuronal death within minutes. In the penumbra, perfusion levels range between 10 and 35 ml/100g/min [4]. Initially, synaptic functioning becomes impaired, while neurons remain structurally intact and viable. If energy supply is restored in time (e.g. by restoration of blood flow), synaptic transmission failure is reversible [4]. However, if oxygen and glucose are not resupplied in time, transition into irreversible damage may occur, and synaptic recovery may be limited, depending on remaining perfusion levels [5,6].

In penumbral tissue with relatively low perfusion levels, close to the infarct core, progression towards irreversible damage is associated with eventual depolarization. This may be preceded by waves of depolarization (‘spreading depression’), leading to cell swelling and -death in the absence of sufficient energy to restore ion gradients [7,8,9]. Otherwise, in areas with mildly disturbed perfusion, mostly located in the outer border of the penumbra, ongoing neuronal damage may continue up to days after the insult and cell swelling may be absent [10].

In conditions with complete ischemia or anoxia synaptic failure is an early consequence of energy depletion [6]. During mild hypoxia, we have shown that isolated synaptic failure may persist during several hours, before progression towards either irreversible damage or recovery [11]. In the penumbra, synaptic failure may be accompanied by production of various proteins (‘heat shock proteins’) [5]. These processes strongly depend on the level of residual flow and the duration of ischemia [5,6]. However, it is incompletely understood which electrical and biochemical processes are vital for recovery and which anticipate permanent damage. For example, suppression of synaptic activity has been proposed as a compensatory mechanism, to balance oxygen supply and consumption in favour of maintaining resting potentials [12], whereas others have shown that suppression of network activity leads to progressive irreversible damage [13,14].

We aim to gain a more profound understanding of neurophysiological processes involved in neuronal recovery or death under conditions of mild to more severe hypoxia / ischemia, such as present in the penumbra. The ultimate goal is to identify potential treatment targets to prevent secondary damage and advance recovery. For this purpose, we use an *in vitro* model system of networks of cortical neurons, cultured on multi electrode arrays, exposed to hypoxia of various duration and depth.

## Methods

### Cell cultures

We obtained cortical cells from new born Wistar rats on the day of birth. After trypsin treatment, cells were dissociated by trituration. We plated about 200,000 dissociated neurons (200 μl suspension) on a multi electrode array (MEA; Multi Channel Systems, Reutlingen, Germany), precoated with poly ethylene imine (PEI). This procedure resulted in an initial cell density of approximately 5000 cells per mm^2^, in agreement with the counted estimates in the first few days after plating. With aging, cell densities gradually decreased to ~2500 cells/mm^2^. These cultures typically contain ~20% inhibitory and ~80 excitatory neurons [15]. We used MEAs containing 60 titanium nitride electrodes with a 30 μm diameter and 200 μm pitch. The electrodes were used for recording, as well as electrical stimulation.

Neurons were cultured in a circular chamber with inner diameter d = 20mm, which was glued on top of the MEA. The culture chamber was filled with ~700 μL R12 medium [16], containing 1.24g/L D-glucose (6,9 mM). MEAs were stored in an incubator, under standard conditions of 37°C, 100% humidity, and 5% CO_2_ in air. For recordings, we firmly sealed the culture chambers with watertight, but CO_2_ and O_2_ permeable, foil (MCS; ALA scientific) and placed the cultures in a measurement setup outside the incubator under a Plexiglas hood (40×40×20 cm). The gas mixture under the hood was continuously refreshed at a flow rate of 4.7 L/min. The oxygen fraction in the refreshing gas mixture was regulated by two computer controlled mass flow controllers. During recordings, we maintained the CO_2_ level of the environment around 5% and we maintained humidity. For details about the recording setup, see [17]. Experiments started at 24.3±1.9 days after plating of the dissociated cells.

All research has been conducted according to Dutch law (as stated in “Wet op de dierproeven”), and approved by DEC, the Dutch Animal Use Committee. To ameliorate animal welfare, parent rats were kept in an IVC system with group housing and environmental enrichment. At the day of birth, donor rats were immediately decapitated after taking them out of the cage, while two pups were kept with the mother.

### Induction of hypoxia and oxygen measurement

To model the limited perfusion of the ischemic penumbra, we aimed to restrict the available amount of adenosinetriphosphate (ATP). Depending on the availability of oxygen, a certain amount of ATP can be produced from glucose. In the penumbra of a brain infarct, the availability of both glucose and oxygen are compromised. In our experimental protocol, we chose to restrict the available amount of oxygen, but not glucose. The available amount of oxygen could easily be changed (up or down) without interrupting recordings or interfering with culture sterility, and still directly affected the available amount of ATP.

Two mass flow controllers were used to obtain three different mixtures of air and N_2_, delivered to the Plexiglas hood at a total flow of 4.7 L/min. Settings (in L/min) were: 4.7 air + 0 N_2_ (normoxia), 2.35 air + 2.35 N_2_ (mild hypoxia) or 0.47 air + 4.23 N_2_ (severe hypoxia). A third flow controller added 0.235 L/min CO_2_ (5%) to any air/N_2_ mixture. In three separate experiments, we recorded partial oxygen pressure (pO_2_) in the culture medium using an optical oxygen sensor (cylindric: Ø = 3.175 mm, l = 63.5 mm; PHOSPOR, Ocean Optics). Before inserting the sensor into the culture chamber, near the neurons, it was calibrated in air (pO_2_ = 20.8 × atmospheric pressure) and N_2_ (pO_2_ = 0). In these experiments we recorded activity before and after the oxygen measurement to confirm that the cultures were alive throughout the period of oxygen measurement. It appeared that insertion of the probe adversely increased noise levels, which hampered reliable simultaneous recording of pO_2_ and electrophysiological signals. Moreover, insertion of the probe challenged sterility during the measurements, which was essential to enable recordings up to several days. Since the oxygen fraction in the air under the hood followed the same steps in all experiments, and the resulting pO_2_ in the medium reproduced very well, results from these experiments were extrapolated and electrophysiological recordings were obtained without simultaneous oxygen measurement.

### Experimental protocol

In all experiments, we recorded spontaneous activity as well as responses to electrical stimulation. Effective electrical stimulation usually induces a response in two phases: a direct response (early phase) and an indirect response (late phase). The direct response contains action potentials of neurons that are close enough to the stimulation electrode to be directly activated by the stimulus pulse, or that have an axon running close enough to the stimulation electrode to be directly activated. This phase of the stimulus response is very reproducible, shows little jitter, and persists during synaptic blockade [18,19], indicating that a substantial part of the response in this phase is not synaptically mediated. The late phase of a stimulus response is abolished after synaptic blockade and represents the indirect, synaptically mediated network response. We will refer to the late phase as synaptically mediated phase.

In all cultures, we first stimulated all electrodes in random order to determine which electrodes were suitable to trigger stimulus responses, and what stimulus strength was required. In all cultures, we selected 2–3 suitable stimulation electrodes that induced clear stimulus responses (including a synaptically mediated phase) before the start of the actual experiment.

Experiments began after an accommodation period of at least 20 minutes. Recordings consisted of a normoxic baseline period of two hours, followed by a period of severe hypoxia of 6, 12, 24, or 48 hours, or mild hypoxia of 48 hours, and at least 3 hours after return to normoxia. Each hour included ten minutes of stimulation of the selected electrodes (in random order, 40 pulses per electrode) and 50 minutes of spontaneous activity.

### Recording and analysis of spontaneous activity

Data were collected from all electrodes at a sample rate of 16 kHz, using a custom program that estimated the noise levels for each electrode in real time. Potential spikes were stored whenever the signal exceeded a predefined threshold of 5.5 times the estimated noise level. Upon each threshold crossing, a peak was determined as the local maximum of the absolute signal. For each spike, the program stored the time stamp of the peak, the recording electrode on which it was detected, and 6 ms of the spike wave shape, from 2 ms before the peak to 4 ms thereafter. Wave shapes were used afterwards for off-line artefact detection [20], and to search for “single neuron electrodes” (see under “Shape of action potentials”).

We studied several parameters in spontaneous recordings: array wide firing rate and functional connectivity as measures of synaptic functioning, and action potential shape as a measure of neuronal integrity. We further differentiated between excitatory and inhibitory neurons based on their firing pattern, as explained in subsection ‘Distinction between excitatory and inhibitory neurons’.

#### Array wide firing rate

The array wide firing rate (AWFR) was defined as the summed activity (total number of action potentials) of all 60 electrodes in bins of 30 minutes. As a control, we continuously measured AWFR in four healthy, normoxic cultures from day 21 to day 24 after plating. Because of the large differences in baseline AWFR between cultures, in all experiment AWFR was normalized to its average baseline value before averaging across experiments.

#### Functional connectivity

Functional connectivity was estimated using a technique to calculate conditional firing probabilities (CFPs) for all pairs of electrodes, adapted from [21]. In short, long term recordings are subdivided into data blocks, each containing a fixed number of action potentials. In each data block CFP_*i*,*j*_[τ] is calculated as the probability to record an action potential at electrode *j* at t = τ, given that electrode *i* detected an action potential at t = 0. CFPs provide a measure for the functional connectivity between two electrodes (*i*,*j*), expressed as a strength (maximum probability; S_*i*,*j*_) and a latency (delay until maximum probability; T_*i*,*j*_). In each data block, the (60×60) connectivity matrix S contains the strengths of the functional connections between all pairs of electrodes. If no functional connection was found between electrodes *i* and *j*, *S*_*i*,*j*_ = 0.

The strength of the functional connections, as expressed by S_*ij*_, is now used to characterize neuronal interactions resulting from synaptic connections. To determine possible changes in the set of connections, we calculated the similarity between connectivity matrices before, during and after hypoxia.

We first looked at functional connectivity strengths. All strengths were normalized to their mean values during baseline. During hypoxia, some functional connections disappeared, whereas other became weaker. Activity usually decreased drastically during hypoxia, and therefore also the number of data blocks (each containing a constant number of action potentials). With the original settings from [21], this sometimes hampered visualization of the development of connectivity during hypoxia. Therefore, we divided long term recordings into smaller data blocks of 10,000 spikes (vs 2^15^ spikes in [21]), and we used a lower threshold for active electrodes of 50 spikes per data block (vs. 250 in [21]). In some cultures with low activity, it was still not possible to obtain a substantial set of at least 30 connections, these were excluded from this type of analysis.

In addition, we investigated whether functional connections that weakened or even disappeared during hypoxia, reappeared after restoration of normoxia. Therefore, we calculated a similarity index between connectivity matrices before, during, and after the hypoxic period as described in [21]. In short: Let *N* be the number of functional connections found in a data block, defined as

$$N=\;\left|\left\{S_{i,j}\neq 0\right\}\right|=\;\left|S\neq 0\right|$$ (1)

Here {*S*_*i*,*j*_ ≠ 0} is the set of electrode pairs (*i*, *j*) with nonzero values in the connectivity matrix *S*. For readability we will use the notation {*S*≠0} for this set of pairs. |{*S* ≠0}| is the cardinality of the set (the number of non-zero elements in *S*).

Similarity indices (SI) between two connectivity matrices *S*_*A*_ and *S*_*B*_, corresponding to data block A and B, were calculated as

$$SI=\;\sqrt{\frac{{\left|\left\{S_{A}\neq 0\right\}\cap \left\{S_{B}\neq 0\right\}\right|}^{2}}{\left|S_{A}\neq 0\left|∙\right|\left\{S_{B}\neq 0\right\}\right|}}$$ (2)

where {*S*_*A*_ ≠ 0} ∩ {*S*_*B*_ ≠ 0}| is the number of pairs that have non-zero value in both connectivity matrices *S*_*A*_ and *S*_*B*_.

#### Shape of action potentials

If hypoxia affects the resting membrane potential, this is also reflected in a change in the action potential shape [22], which can be detected in extracellular potential recordings [23]. We defined spike activity recorded from a single electrode as single neuron action potentials if the shapes of a sufficient number of action potentials (at least 100 during the first hour of baseline recordings) were sufficiently similar during baseline, and if there were no (1 ms) refractory period violations.

Similarity of action potentials was quantified by analysis of the variability of recorded signals from 1.5 ms before to 1.7 ms after the peak of each detected action potential. From the available 96 samples (6 ms, sampled at 16 kHz) per recorded action potential, we used samples 9–59 to calculate the mean (Mean_AP_ [n]) and standard deviation (SD_AP_[n]) of each sample n (9≤n ≤59). We calculated a measure for variability:

variability=|∑n=959SDAP[n]∙MeanAP[n]51|max{|MeanAP|} (3)

where max{|*Mean*_*AP*_|} represents the amplitude of the averaged action potential shape. The numerator is chosen such that emphasis is given to action potential shape differences that appear near the peak of the action potential. Activity from an electrode with *variability* < 1 was considered as single neuron activity.

For those electrodes that passed this threshold, we studied the temporal evolution of the shape of the action potential during and after the hypoxic period. First, we determined the average action potential shapes of these electrodes per 1h time bin, in all bins that contained at least 5 action potentials. Then, we calculated the mean correlation coefficient between the average action potential shape in a certain hour and the average action potential shapes of all preceding hours. As long as the shape of an action potential remained constant, the average correlation coefficient was close to one. Because the correlation coefficient is sensitive only to relative shape differences, but not to absolute scaling, we also monitored the action potential amplitude. The shape of an action potential was considered constant as long as the correlation coefficient remained above 0.9 and the amplitude did not drop below 80% of its baseline value.

#### Excitatory and inhibitory neurons

To determine if excitatory and inhibitory neurons are differentially affected during hypoxia, we calculated the Fano factors (standard deviation / mean) of spike counts in 6 s bins.[24]. Excitatory neurons were shown to have a Fano factor of 3.17 ±2.02, whereas the Fano factor of inhibitory neurons averaged 29.37±8.45 [24]. We set the threshold to qualify a neuron as excitatory to Fano factor<7.21 (= 3.17+2∙2.02), and qualified a neuron as inhibitory if Fano factor>12.47 (= 29.37–2∙8.45). Electrodes with values in between were classified as unknown. For all neurons that could be classified as excitatory or inhibitory, we determined whether they became inactive before the end of the experiment, and whether or not the shape of their action potential changed. We interpreted a changing action potential shape shortly before becoming inactive as loss of membrane integrity. Neurons that became inactive without changing action potential shape might also have lost excitatory inputs.

### Induced activity by electrical stimulation

Additionally, we evaluated the responses to electrical stimulation (biphasic current pulses, 200 μs perphase, 12–32 μA). At these amplitudes electrolysis is avoided and no noticeable damage inflicted on the neurons. Inter pulse intervals were chosen between 5 and 10 s, such that subsequent stimulus responses showed no decline. At the beginning of each experiment, we selected 2–3 electrodes for stimulation during the first 10 minutes of every hour.

For each stimulation period, we calculated the average response to stimulation of each electrode, and quantified the synaptically mediated phase by the area under the curve from 15–150 ms (A_*Syn*_). We did not analyse the early phase of stimulus responses because these often contained a mixture of directly induced and synaptically mediated action potentials. To enable comparison between experiments, for each electrode *A*_*Syn*_ was normalized to its baseline value. To avoid unstable normalization, we set a threshold for *A*_*syn*_(baseline) at 6 spikes.

### Life/death assay

In parallel to the cultures plated on MEAs, we plated cells on glass coverslips which were stored in the incubator under conditions as described above for 3 weeks to allow for network maturation. These cultures were exposed to severe hypoxia (pO_2_≈20mmHg) for 0h (baseline, n = 2), 6h (n = 2), 24h (n = 2), or 48h (n = 2) before washing with cold PBS and incubating with a 100 μl /ml solution of propidium iodide (PI) for 15 minutes at room temperature. Subsequently, cells were washed with binding buffer and fixated in formalin for 10 min. Then cell membranes were permeabilized using 0,2% Triton-X100 for 5 min, in the presence of 600 nM 4',6-diamidino-2-phenylindole (DAPI) in PBS for 10 minutes. After washing with PBS, samples were mounted with mounting medium (Mowiol, Sigma-Aldrich). Images were obtained using a Nikon DS-F*i*1 digital camera linked to an upright fluorescence microscope (Nikon Eclipse 50*i*) with a 20 x objective.

### Statistical analysis

Two-way ANOVA was applied to assess the statistical significance of differences between pre-, peri- and post-hypoxia parameter values. We used the Kruskal-Wallis test to determine a possible trend in the temporal development of AWFR of normoxic cultures. P<0.05 was considered statistically significant. All results are shown as the mean ± the standard error of the mean (SEM), unless indicated otherwise.

## Results

### Induction of hypoxia

**Fig 1:**
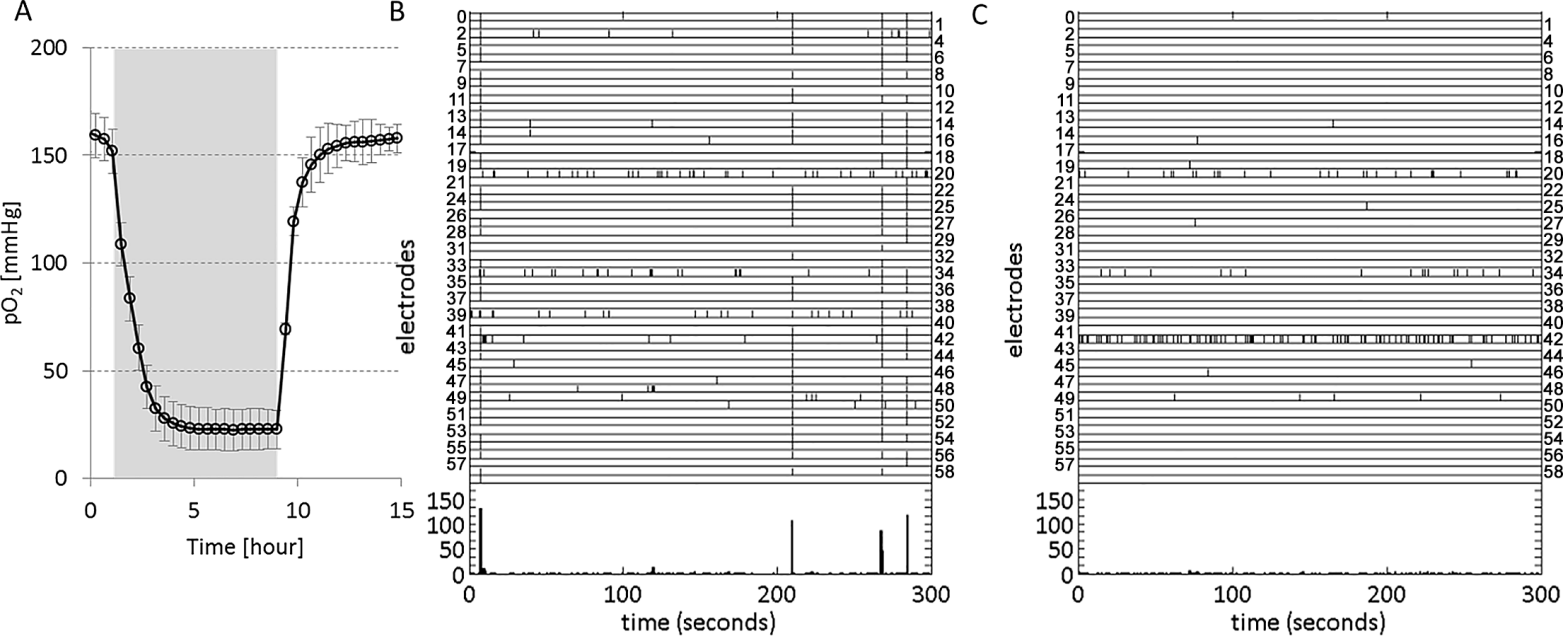
Temporal evolution of the partial oxygen pressure (pO_2_) in an MEA filled with R12 at 36°C, containing a cortical culture and raster plots during normoxia and hypoxia. **A**: pO_2_ was measured in an MEA with a cortical culture, placed under a Plexiglas hood that received a continuous gas flow of 4.7 L/min. The gas mixture was either normoxic (95% air and 5% CO_2_), or hypoxic (85% N_2_, 10% air and 5% CO). Filled background indicates period of hypoxic gas flow, referred to as hypoxic period. Curve shows the average of 3 measurements, error bars indicate SD. **B, C:** Activity raster plots during normoxia (A) and hypoxia (B), recorded in one culture. Vertical axes indicate electrode numbers, each tick marks an action potential at the electrode number indicated on the left or right of the row. The bottom part of both panels shows total activity in 1 s bins. Under normoxic conditions, synchronous network bursts were regularly observed (e.g. at t = 10s or t = 210s in panel B). Under hypoxic conditions, typically the array wide firing rate decreased, as well as the synchronicity between electrodes. Occasionally, a small number of neurons (<5) displayed increased, uncorrelated firing, e.g. electrode 42 in panel C.

In three cultures we measured the partial oxygen pressure in the culture bath. A baseline pO_2_ of 160 mmHg dropped to 23 mmHg with a time constant of 0.95±0.04 (SD) hour upon switching to the hypoxic gas mixture of 4.23 L/min N_2_ and 0.47 L/min air, and it returned to pO_2_ = 160mmHg with a time constant of 0.51±0.10 (SD) hour after return to normoxic gas supply (4.7 L/min air), see Fig 1A. Switching to a hypoxic gas mixture of 47.5% N_2_, 47.5% air and 5% CO_2_ yielded a similar response, with a plateau at pO_2_ ≈ 80 mmHg.

### Array wide firing rate

**Fig 2:**
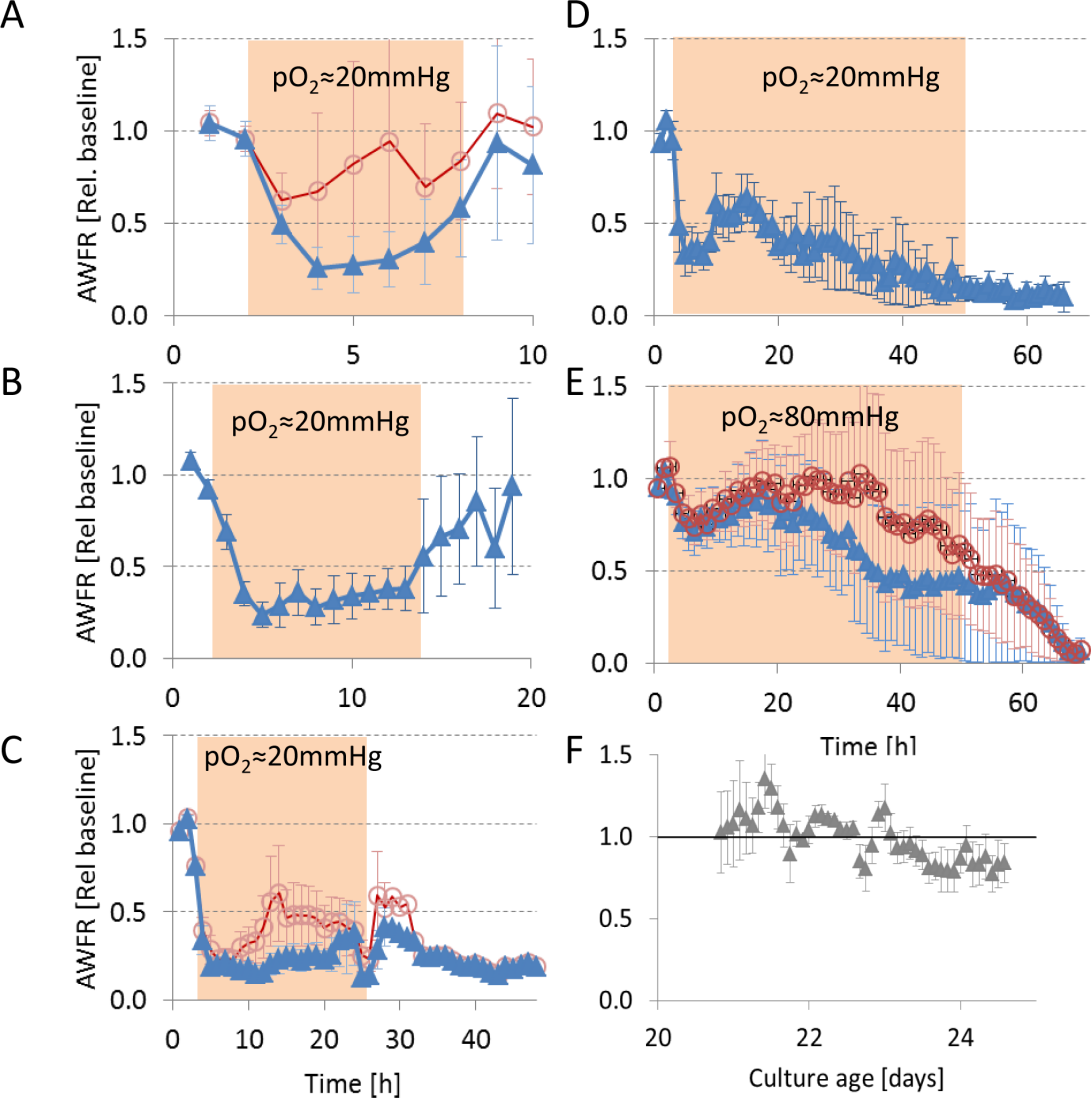
Temporal evolution of array wide firing rate (AWFR) in cultures exposed to hypoxia (pO_2_≈20 mmHg) for 6 (A), 12 (B), 24 (C), or 48 hours (D), and 48 hours at pO_2_≈80 mmHg (E). Filled background areas indicate the hypoxic period. All curves show a clear decrease of AWFR at the start of the hypoxic period followed by complete recovery after 6 or 12 hours. During longer hypoxic periods this initial decrease was followed by partial recovery up to ~18 hours. In four experiments, a few electrodes showed significantly increased activity and dominated AWFR (see text). Triangles indicate averages after removal of these outliers, circles show the average of all data. Error bars indicate SEM. Panel F shows the temporal development of AWFR in normoxic periods of 72 hours, recorded between 21 and 24 days after plating, as a control.

In control recordings of four normoxic cultures during the period 21–24 days after plating (72 hours), mean AWFR fluctuated around a constant level without a significant trend (Kruskal Wallis, p>0.99, see Fig 2F).

We obtained activity measurements from 21 cultures exposed to severe hypoxia (pO_2_≈20mmHg) for 6 hours (n = 4), 12 (n = 5), 24 (n = 9; total recording ≥ 29 hours: n = 4) or 48 hours (n = 3). In all experiments AWFR immediately decreased with the onset of the hypoxic period, in most experiments AWFR remained below baseline during the entire hypoxic period. Fig 1B and 1C show two representative raster plots, one under normoxic and one under hypoxic conditions. Besides decreased AWFR, typically, synchronicity between electrodes also decreased.

Mean activity during hypoxia was lower than during baseline in all groups (p<0.001), except for the 6 hour group (p = 0.22) (Fig 2). In four experiments (one 6-hour, two 24-hour and one 48 hour experiment), the initial decrease in AWFR was followed by an increase to values above baseline before the end of the hypoxic period. In all cases, this resulted from increased activity from 1–3 electrodes only, while activity at all other electrodes was substantially lower than during baseline. This focally increased activity showed little or no synchronization with other electrodes, and dropped back to baseline levels after the hypoxic period in all four experiments. After exclusion of the experiments with focally increased activity, the decrease of AWFR during hypoxia reached statistical significance for all durations (p<0.001). AWFR recovered to baseline values after 6 or 12 hours (p>0.11), but not after 24 or 48 hours of hypoxia (p≤0.0004).

Under less severe hypoxic conditions AWFR remained around baseline level until 30 hours of hypoxia (n = 4). However, the average firing rate was dominated by one experiment with focally increased activity at two electrodes. Average activity on all other electrodes dropped to 50% of baseline after 30 hours.

### Functional connectivity

We investigated the effect of hypoxia on the strengths of functional connections (how strongly are two electrodes connected) and on the set of connections (which electrodes are connected).

**Fig 3:**
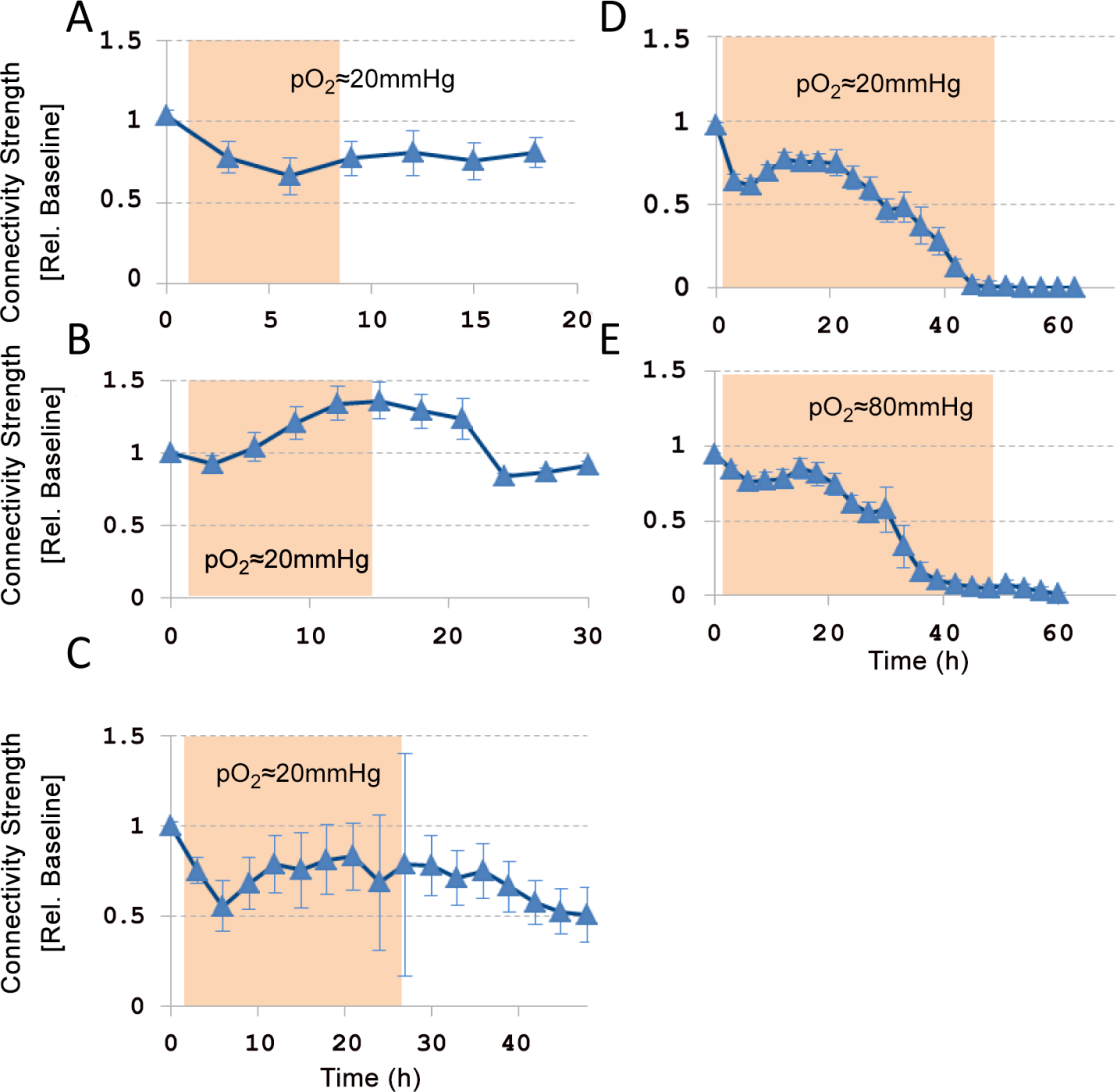
Temporal evolution of functional connection strength during 6 (n = 4 cultures), 12 (n = 3), 24 (n = 4), or 48 (n = 4) hours of hypoxia at pO_2_≈20 mmHg, or during 48 hours at pO_2_≈80 mmHg (n = 3). The strengths of all functional connections were normalized to their baseline values. Error bars show SEM and reflect differences between cultures. Filled background indicates hypoxic period.

Strength of functional connections: Functional connectivity could be determined before, during, and after hypoxic periods at pO_2_≈20 mmHg of 6h (n = 4 cultures), 12 h (n = 3), 24 h (n = 7; post-hypoxic period >3h: n = 4), or 48 h (n = 3), and in three cultures that were exposed to 48 hours of hypoxia at pO_2_≈80mmHg. Four cultures were excluded because activity during baseline was insufficient for the statistical analysis required to adequately determine functional connectivity, yielding less than 30 connections. The included cultures had 236±50 connections during baseline on average. The strength of functional connections always decreased during the first 3–6 hours of hypoxia. During continuing hypoxia, this initial decrease was followed by partial recovery within the next 15–20 hours, as indicated by the positive slope in this period. If the oxygen supply was restored after 6, 12, or 24 hours, average connection strengths recovered. However, after 24 hours of hypoxia, large differences between functional connection strengths were observed between cultures. Some connection strengths had increased to levels above baseline, whereas other had dropped to zero. After 48 hours of hypoxia, there was no recovery of functional connections, independent of hypoxic depth (Fig 3).

**Fig 4:**
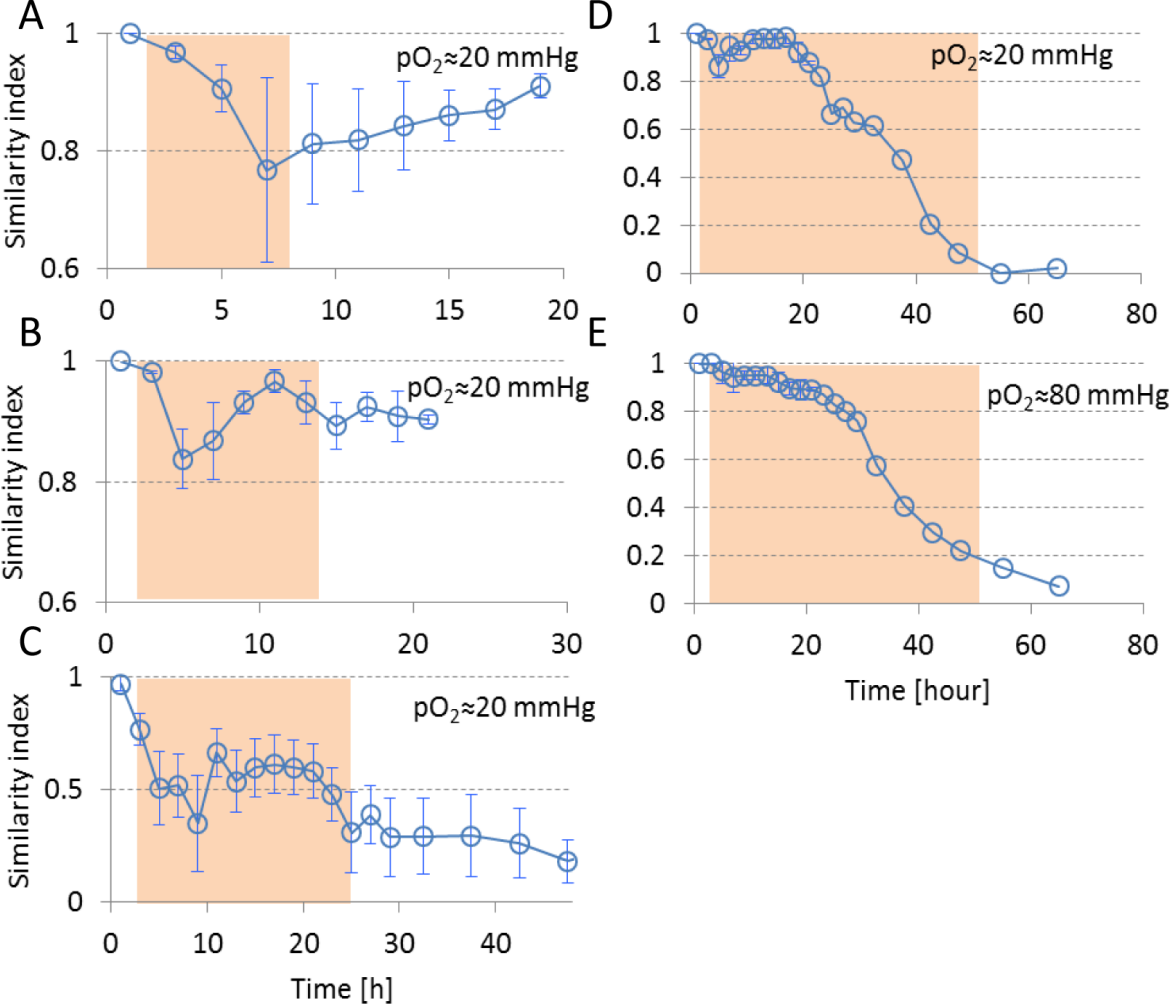
Temporal evolution of similarity index (Eq 2) before, during, and after hypoxia. Average values are shown for 2 hour time bins. Errorbars indicate SEM and reflect differences between cultures. Filled backgrounds mark the hypoxic (pO_2_≈20 mmHg or pO_2_≈80mmHg) period.

Sets of functional connections: Besides strengths of functional connections, we investigated possible changes in the sets of connections during and after hypoxia, using the similarity index. Fig 4 shows the development of the similarity index before, during, and after hypoxia. During the first 4–8 hours of the hypoxic period, similarity decreased, mainly due to disappearing functional connections. If the oxygen supply was restored within 12 hours, similarity to baseline connectivity largely recovered (SI≥0.9). During continuing hypoxia, similarity to baseline connectivity still largely recovered in the period 6–18 hours (SI>0.85), indicating that old connections recovered and that recovery of the average connection strength did not involve the formation of new connections. After ~20 hours, changes became irreversible, restoration of the oxygen supply no longer yielded recovery of similarity to baseline connectivity (SI<0.5), although several connections remained active and the average connectivity strength hardly decreased. After ~40 hours of hypoxia similarity to baseline connectivity was irreversibly lost.

### Neuronal integrity of excitatory and inhibitory neurons

**Fig 5:**
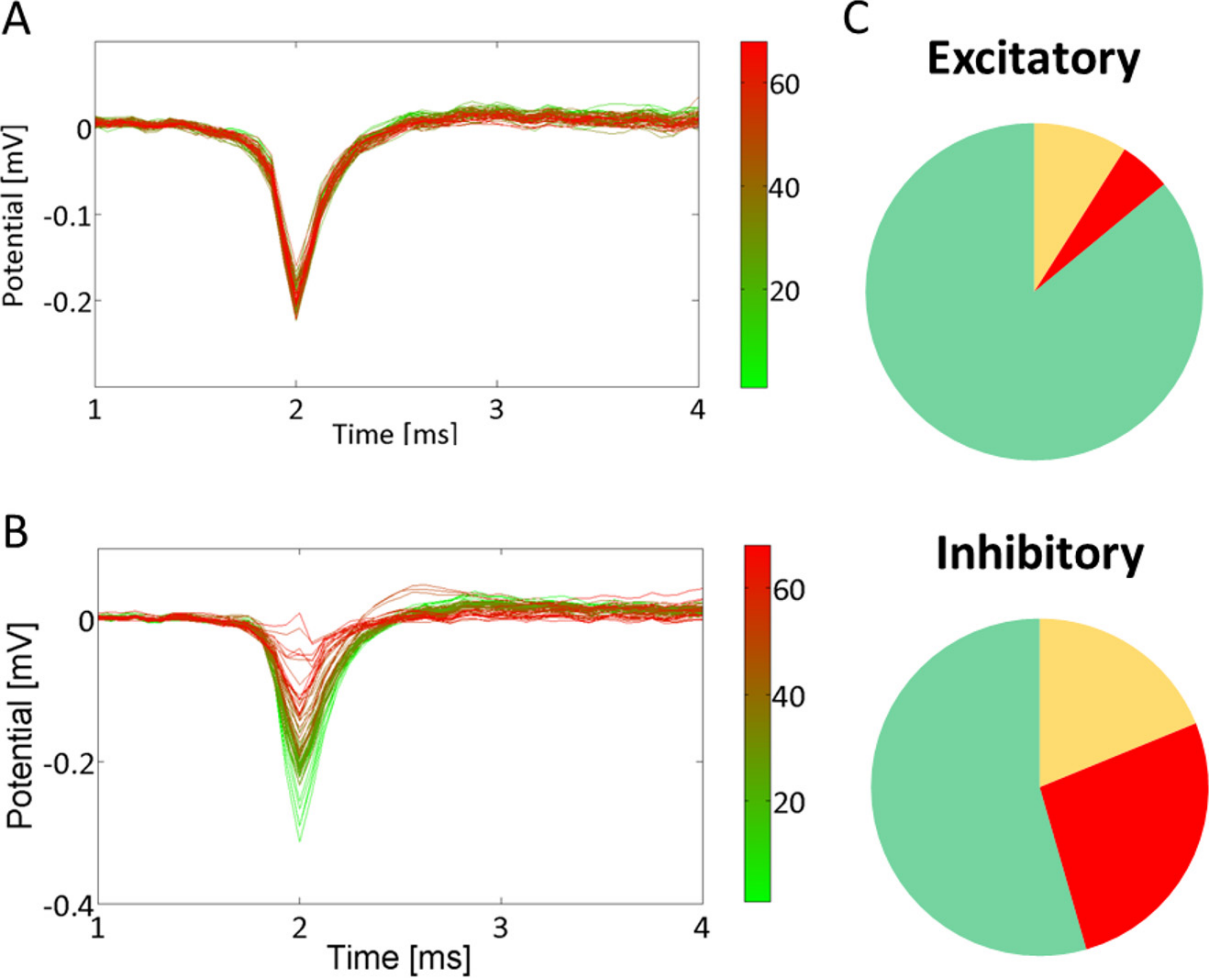
Action potential shapes. A, B: Mean action potential shape per hour of two electrodes that recorded single neuron activity. Several neurons stopped firing during the experiment, some after the shape of the action potential had changed, some without action potential shape change. B was classified as a changing action potential shape, A was not. Colours encode the hour of averaging, as indicated in the colour maps. C: Several single neuron electrodes could be classified as excitatory or inhibitory. The upper panel shows the fraction of all excitatory neurons that remained active throughout experiments (green), and the fractions that became inactive with (red) or without (yellow) a changing action potential shape. The lower panel shows these fractions of all inhibitory neurons.

In 19 experiments at pO_2_≈20 mmHg, we found 93 single neuron electrodes. 44 could be classified as excitatory (Fano Factor<7.2) and 11 as inhibitory (Fano factor>12.5). 69 (74%) of these single units remained active throughout the experiment with unchanged action potential shape and amplitude (Fig 5A). The other 24 stopped firing during (18) or after (6) the hypoxic period. Sixteen of these 24 neurons showed a changing action potential shape (see Fig 5B) before their activity dropped to zero, indicating membrane failure. If the shape of the action potential changed, this occurred after 27±5 hours of hypoxia. Stopping of firing occurred in 14% of all excitatory and 45% of all inhibitory neurons. Preceding changes of action potential shape occurred in 5% of all excitatory and 27% of all inhibitory neurons (Fig 5C). Action potential shapes of inhibitory neurons remained intact for at least 16 hours of hypoxia, those of excitatory neurons for at least 18 hours.

### Responses to electrical stimulation

**Fig 6:**
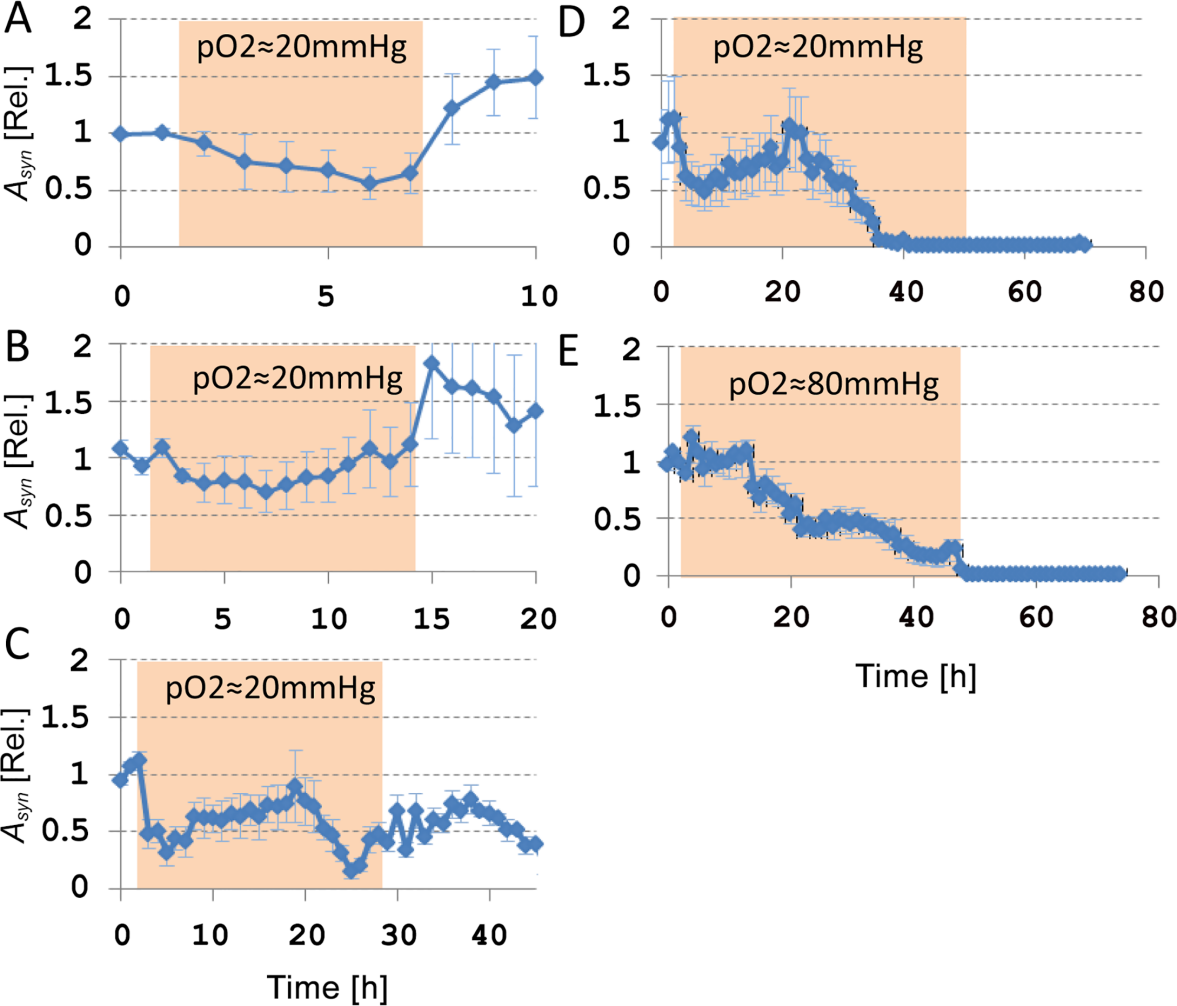
Temporal evolution of the network responses to electrical stimulation before, during, and after 6 hours of hypoxia at pO_2_≈20mmHg (9 stimulation electrodes in 4 different cultures), 12 hours (11 electrodes, 6 cultures), 24 hours (15 electrodes, 5 cultures), or 48 hours (12 electrodes, 4 cultures), or 48 hours at pO2≈80mmHg (6 electrodes, 3 cultures). *A*_*syn*_: area under curve of the synaptically mediated stimulus response. Before averaging across experiments, we normalized *A*_*syn*_ per electrode per culture to its mean baseline value. Filled backgrounds mark the hypoxic periods. Error bars indicate SEM.

Fig 6 shows the development of the synaptically mediated network responses to electrical stimulation, quantified by *A*_*syn*_, before, during, and after 6 (n = 9 electrodes in 4 cultures), 12 (n = 11 electrodes, 5 cultures), 24 (n = 20 electrodes, 8 cultures; post hypoxic recording > 3h: n = 8 electrodes, 3 cultures), or 48 hours (n = 9 electrodes, 3 cultures) of hypoxia at pO_2_≈20mmHg. In all experiments, *A*_*syn*_ decreased to approximately 50% of its baseline value during the first 6 hours of hypoxia. Mean *A*_*syn*_ was lower during the hypoxic period than during baseline (ANOVA, p<0.03). If the oxygen supply was restored after 6 hours, *A*_*syn*_ completely recovered to baseline values (ANOVA, p = 0.96). In longer hypoxic periods, the initial decrease of *A*_*syn*_ was usually followed by recovery during hour 6–20 to values close to baseline. Return to normoxia after 12–24 hours yielded some recovery of *A*_*syn*_, as compared with the lowest values during hypoxia, but *A*_*syn*_ remained below baseline levels (ANOVA, p<0.02). Between 30 and 40 hours of hypoxia, *A*_*syn*_ dropped to zero, and restoration of the oxygen supply beyond this point no longer led to persistent recovery.

### Life/ Death assay

**Fig 7:**
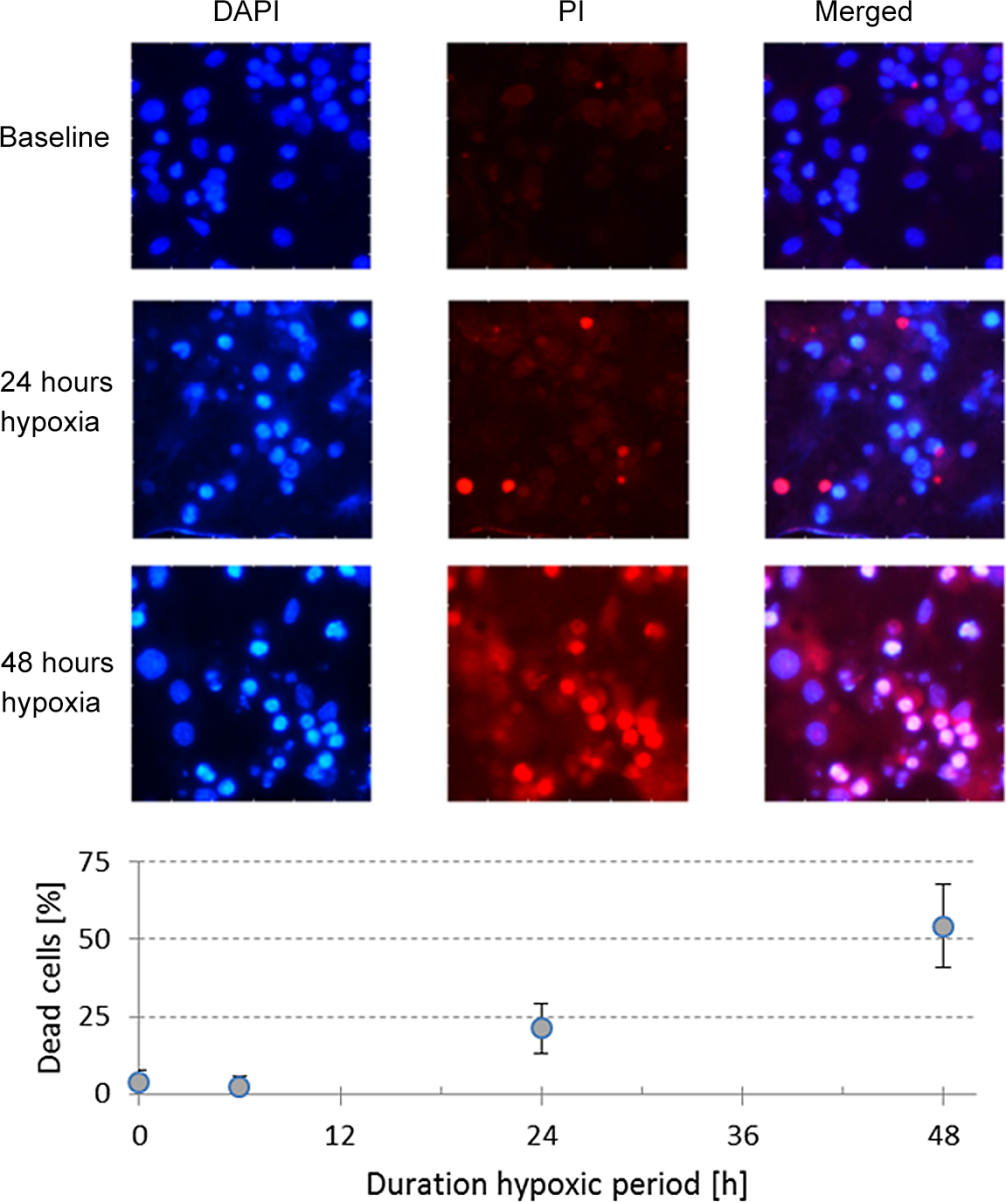
Fraction of dead cells depended on the duration of the hypoxic period. We applied a DAPI staining (blue) to enable counting of all cells in 100×100μm areas, combined with propidium iodide (PI, red) to count the number of dead cells. **A**: Typical examples after 0h, 24h, or 48h of severe hypoxia (pO_2_≈20mmHg). **B**: Percentage of dead cells after 0h (baseline, n = 2 cultures), 6h (n = 2), 24h (n = 2) or 48 h (n = 2) of severe hypoxia. Curve shows the average and SD of 9 +9 images (100×100μm) from both cultures for each hypoxic duration.

Eight cultures plated on glass coverslips were stained to determine the fraction of dead cells before (n = 2), and 6h (n = 2), 24h (n = 2) and 48h (n = 2) after the onset of severe hypoxia (pO_2_≈20mmHg). Under baseline conditions and after 6 hours of hypoxia, cultures typically showed less than 10% of dead cells. This fraction was slightly higher after 24 hours of hypoxia (≈20%), and substantially higher (>50%) after 48 hours. Fig 7 shows typical examples of stained cultures after 0h, 24 h and 48 hours, as well as the average percentages of dead cells, as determined from eighteen 100×100μm images obtained from 2 different cultures per condition.

## Discussion

We investigated the effect of partial hypoxia of varying duration on synaptic and neuronal functioning in cultured neuronal networks. We show an initial, reversible decrease in activity and connectivity, followed by partial recovery up to ≈18 hours of hypoxia. Beyond 18 hours, connectivity changes became irreversible, but neuronal integrity initially remained intact. After 30–40 hours of hypoxia induced neuronal inactivity, irreversible neuronal silence was accompanied by membrane depolarization, at both evaluated depths of hypoxia (pO_2_≈20 or 80 mmHg). These various changes will now be discussed in more detail.

### Initial reversible decrease in activity and connectivity

Six hours of hypoxia impeded synaptic connectivity, but did not affect neuronal integrity. Most neurons remained active throughout the 6 hour hypoxic periods and shape and amplitude of their action potentials did not change. However, connectivity was reduced, resulting in 50% lower responses to electrical stimulation (*A*_*syn*_<0.5), and lower firing rates (AWFR < 50% of baseline). Functional connectivity usually decreased during this period as several connections became weaker or inactive, presumably caused by synaptic failure. However, our use of indirect measures of connectivity and membrane properties preclude a sure distinction with disturbed axonal action potential propagation. Previous analysis suggested synaptic dysfunction as the most probable cause [11]. This would be in agreement with the notion that ATP shortage during hypoxia reduces neurotransmitter release. Reduced neurotransmitter release is associated with less phosphorylation of the synapsin-I protein [25], thus hampering the fusion of vesicles with neurotransmitter with the plasma membrane. All changes were completely reversible, if oxygen was restored at 6 hours.

### Partial recovery

During persistent hypoxia, activity, connectivity and stimulus responses partially recovered between 6 and 18 hours. This suggests partial restoration of synaptic transmission, possibly reflecting some compensatory mechanism. Under healthy conditions, homeostatic activity regulation occurs by synaptic scaling; when electrical activity is below a set-point, excitatory synapses are up-regulated [26], and inhibitory synapses are down regulated [27]. First signs of local synaptic scaling occur after 4–6 hours [26] and have an immediate effect on postsynaptic firing rates, which is in agreement with our observations. Global synaptic scaling takes place over a time period of 24–48 hours [26]. Up regulation of excitatory synapses could explain the increased functional connectivity in the period between 6 and 18 hours (Fig 3). Synaptic scaling would require potentiation of glutamatergic synapses. Increased phosphorylation of the α-amino acid tyrosine may potentiate NMDA [28], as well as AMPA currents [29]. Despite ATP scarcity, increased tyrosine phosphorylation has indeed been observed under hypoxic conditions [25], supporting the view of inactivity induced synaptic scaling. Activity homeostasis also controls the growth of axons [30] and dendrites [31], and the formation of spines and boutons [32]. However, this mechanism operates on time scales that are substantially longer than the 6–18 hours observed in our study [33].

In the 12 hour hypoxia group, functional connectivity even increased to values above baseline during the hypoxic period. This unexpected, given the general impression that synaptic efficacy decreased during hypoxia (*A*_*syn*_ and AWFR decreased by 50% in these experiments). This average functional connectivity above baseline mainly resulted from one experiment with relatively few connections (40, vs. 180 connections on average in the other experiments in this group), and the experiment-wise averaging of Fig 3 probably overemphasized the actual connectivity strength. Although it remains unclear why they became stronger than during baseline in this experiment, it underlines the generally observed recovery of functional connectivity between six and eighteen hours.

If oxygen was restored after 12 hours, all changes were reversible and network connectivity returned to baseline. Similarity between pre- and post-hypoxic connectivity matrices was around 0.9. Similar values were found in 3–4 weeks old cultures under normoxic conditions [21].

### Irreversible connectivity changes with intact neuronal integrity

Beyond ~18 hours of hypoxia, AWFR, *A*_*syn*,_ and functional connectivity decreased again, while the number of neurons that ceased firing increased. Neurons can stop firing due to e.g. membranedepolarization or insufficient excitatory input. Both may cause subsequent cell death^,^[13,14]. In healthy neurons the shape of recorded action potentials is very stable [34], and a suddenly changing shape of the action potential may reflect membrane failure. As opposed to within 18 hours, if neurons became inactive in the period beyond 18 hours, the shape of the action potential often changed first. This suggests that beyond 18 hours most neurons became inactive due to membrane failure. This is supported by staining experiments that showed that cell death predominantly occurred after 24–48 hours, and by the observation that beyond 18 hours changes were no longer completely reversible, although many neurons were still alive.

### Irreversible neuronal silence

After 30–40 hours at pO_2_≈20mmHg, *A*_*syn*_ and functional connectivity dropped to zero, and AWFR dropped to zero in all but one experiment. These changes were irreversible and probably the consequence of massive cell death. During mild hypoxia (pO_2_ = 80mmHg), these changes occurred after approximately the same time interval. It seems unlikely that neuronal cell death occurred predominantly as a direct consequence of failure of Na^+^/K^+^-ATPase, because in that case neurons would be expected to survive longer with higher levels of oxygen. Since average activity was far below baseline under both conditions of hypoxia, irreversible neuronal silence was caused probably by the preceding period of low activity, rather than oxygen depletion after 30–40 hours. Insufficient activity leads to elimination of synapses[35] and cell death, modulated by calcium dependent mechanisms that may involve Myocite Enhancing Factor 2 (MEF2)[14] or Brain Derived Neurotrophic Factor[36] (BDNF).

### Distinct effect on excitatory and inhibitory neurons

In four out of twenty-one experiments, total activity increased during hypoxia, caused by 1–3 electrodes with impressively increased activity. In contrast to the activity patterns at most electrodes, these electrodes picked up activity from tonically firing neurons, suggesting that they were intrinsically active. The accompanying low values of *A*_*syn*_ and functional connectivity strength indicated largely decreased synaptic functioning. Probably, the high activity at these three electrodes resulted from disinhibition of intrinsically active neurons, which have been found in various layers of the cortex [37]. This disinhibition might be due to failure of inhibitory synapses, which would be in contrast to previous findings that suggested that inhibitory synapses are less vulnerability to hypoxia than excitatory synapses [38]. Alternatively, the increased activity may result from selective cell death of inhibitory neurons [39] or failure of glutamatergic synapses on inhibitory cortical interneurons [40].

To investigate possible differences between vulnerability of excitatory and inhibitory neurons, we used the Fano factor of spike counts at electrodes with presumed single neuron activity. This technique was adapted from Becchetti et al [24], who showed that the Fano factor of spike counts was the best discriminator in cultures of mice cortical neurons. In the current study we relied on their findings with no further confirmation than the ratio of excitatory to inhibitory neurons thus obtained being 4:1,which is in good agreement with many experimental studies (see for example [15]). 14% of the excitatory neurons stopped firing before the end of the experiment versus 45% of the inhibitory neurons. The relatively large fraction of inhibitory neurons with affected action potential shape just before they became inactive suggests a higher vulnerability as a plausible explanation.

### *In vitro* model of the penumbra

An important advantage of our *in vitro* model system is that, unlike acute brain slices, cultured neuronal networks can stay alive for extended periods of time, which enabled us to do experiments that lasted up to three days. The most prominent feature that defines the difference between the penumbra and the infarct core is the initial absence of large scale cell death. Staining experiments confirmed that massive cell death first occurred between 24 and 48 hours after the onset of severe hypoxia. The applied staining was not specific to neurons and included glial cells. Still, most dead cells were probably neurons because glial cells are less vulnerability to hypoxia than neurons [41]. Multi electrode arrays provide extracellular recordings of neuronal activity. The non-invasive nature of this technique contributes to the long term vitality of cultures, but carries the disadvantage that it does not enable direct measurement of membrane potentials. Consequently, we had to rely on the shape of the action potentials to provide an indirect measure of neuronal integrity. This indirect measure may have masked subtle changes in the resting membrane potential.

A potential limitation of the use of cultured neuronal networks is the lack of normal brain architecture. However, the focus of this study was on general synaptic and neuronal functioning during hypoxia, which does not require a specific brain structure. Furthermore, the relatively small number of neurons in our model system and the monolayer topology may result in different time scales of progression of neuronal damage, e.g. due to a lower number of synaptic contacts [42] or diffusion processes that occur at a slower pace than ischemia during stroke *in vivo*. After brief bilateral carotid artery occlusion in gerbils, Nair et al found an oxygen disappearance rate of 24mmHg/s [43], which is much faster than the disappearance rate in our study. However, this will most likely not affect the qualitative findings as reported in our study.

Another possible limitation lies in the interpretation of oxygen levels and the definitions of normoxia and hypoxia. Normoxia in the *in vivo* rat brain has been reported at pO_2_≈30–35mmHg [43,44], five times lower than normoxia in our study, and even 50% lower than the mildest hypoxia level used in our study. Networks of dissociated cortical neurons are usually cultured under high “normoxic” conditions and it is unclear how this relatively high pO_2_ under *in vitro* conditions relates to physiological oxygen pressure *in vivo*. Still, all cultures responded immediately to decreasing pO_2_, far before it dropped below 30–35mmHg, indicating a clear effect of pO_2_ variations in the applied range. Although the remaining oxygen levels in the experiments cannot be directly related to those in the penumbra of stroke patients, our findings of reduced connectivity and intact neuronal functioning are in agreement with these clinical observations. In the penumbra of a brain infarct, the availability of both glucose and oxygen are compromised. For practical reasons, we chose to restrict the available amount of oxygen, but not glucose (see Methods). *In vivo*, additional processes, possibly related to the limited availability of glucose or activation of other cell components, may occur in parallel to the processes observed in our model system. Other possible differences between the *in vivo* penumbra and our model system may result from environmental factors like the suddenly vanished input from the infarct core.

## Conclusion

In cultured neuronal networks hypoxia up to 6 hours results in reduced neuronal activity due to impeded synaptic transmission. After 6 hours, a compensatory mechanism, presumably to achieve activity homeostasis, is started, leading to partial restoration of activity and connectivity, even if oxygen remains low. If normoxia is restored within 12 hours, changes in network connectivity are largely reversible. If the hypoxic conditions persist, activity levels further drop and connectivity irreversibly lost. As this transition appears independent of remaining levels of oxygen, we speculate that irreversible damage (at least partially) results from insufficient neuronal activation. This opens avenues for testing therapies to improve recovery by neuronal stimulation, for instance using carbachol [45] or ghrelin [46,47].
